# Supplementary material for: Recommendations for Diagnosis and Treatment of Lumbosacral Radicular Pain: A Systematic Review of Clinical Practice Guidelines
Source: J Clin Med. 2021 Jun 3;10(11):2482. doi: 10.3390/jcm10112482 (PMC8200038; doi:10.3390/jcm10112482)
Supplement: Supplementary file 1 [file jcm-10-02482-s001.zip › jcm-1238384-SI.pdf]

Journal of
Clinical Medicine

MDPI

Supplementary Materials

Table S4. Diagnostic Recommendations from guidelines for lumbar radicular pain.

Physical examination	Guideline*	Specifications	Consistency	Clinical Inference
Femoral stretch test	NASS	Insufficient evidence for recommendation	inconsitent	none
	NBPN	Suggested		
	PARM	Suggested		
Straight leg test	NASS	Recommended	Consistent	Should do
	PARM	Recommended		
	IHM	Strong recommendation		
	NHG	Important to do		
	ACP-APS	Should be done		
	NBPN	Recommended		
Crossed straight leg test	NASS	Recommended	consistent	Should do
	PARM	Recommended		
	IHM	Strong recommendation, high specificity and low sensibility		
	NHG	Important to do		
	NBPN	Recommended		
Muscle testing	NASS	Recommended	Common/consistent	Should/could do
	PARM	Suggested: knee extension, foot dorsi and plantarflexion, toe dorsiflexion		
	IHM	Recommended; foot plantar and dorsiflexion, knee extension strength, toes dorsal flexion		
	NHG	Important to do		

Journal of Clinical Medicine

	ACP-APS	should be done; knee, great toe and foot dorsiflexion		
	NBPN	Strong recommendation		
Sensory testing	NASS	Suggested; foot sensibility	Common	Could do
	PARM	Recommended		
	IHM	Should be carefully considered; foot		
	ACP-APS	Should be done; distribution of sensory symptoms		
	NBPN	Weak evidence		
Reflex tests (ankle and knee tendon)	PARM	Suggested	Inconsistent	None
	NBPN	Weak evidence		
	ACP-APS	Recommended		
	NASS	insufficient evidence to make a recommendation		
mapping pain distribution	PARM	Recommended	Consistent	Should do
	IHM	Strong recommendation		
Slump test	PARM	Suggested	inconsistent	None
	NASS	Insufficient evidence to make a recommendation		
Wasserman test	PARM	Suggested	Consistent	Could do
	IHM	Should be carefully considered		
Steppage	PARM	Recommended; Immediate referral for surgical evaluation	Consistent	Should do
	IHM	Recommended; requires immediate surgical evaluation		

Journal of
Clinical Medicine

Congruence of signs and symptoms	PARM	Recommended	Consistent	Should do
	IHM	Strong recommendation		
Diagnostics<br>Imaging<br>*Routinely offering imaging in primary care or absent of red flags*	KCE	Do not offer	Consistent	Do not do
	ICSI	Should not routinely recommend		
	NICE	Do not offer In a non-specialist setting, consider in specialist settings		
	ACOEM	Routine CT not recommended		
	CCGI	Avoid unless serious underlying pathology suspected		
	ACR	CT/MRI/X-ray/X-ray myelography usually not appropriate if no prior management		
	NBPN	Do not offer		
Computed Tomography (CT)/ Magnetic resonance imaging (MRI) routinely in first 4–6 weeks	ICSI	Should not routinely recommend	Consistent	Do not do
	ACOEM	Not recommended		
	NHG	advice against in primary care		
	PRAM	Not recommended unless highly painful sciatica or progressive motor deficit		
	IHM	Not recommended if no highly-painful sciatica or progressive motor deficit		
	NBPN	Do not offer		

Journal of *Clinical Medicine*

CT when history and physical examination findings consistent with disc herniation, after 4 – 6 weeks of low back pain if surgery is considered, severe or progressive neurologic signs and symptoms present	NASS	CT/CT myelography recommended	Consistent	Should do
	VA/Dod	Recommended		
	TOP	If MRI is contraindicated		
	PRAM	Strongly endorsed		
	ACOEM	Recommended		
	IHM	Recommended		
	ACP-APS	Only if patients are potential candidates for surgery or epidural steroid injection		
	NBPN	If MRI is contraindicated		
	ACR	Usually appropriate when underlying diseases present<br>May be appropriate when surgery or intervention candidate		
MRI when history and physical examination findings consistent with disc herniation, radiculopathy persists after six week, if surgery is considered, severe or progressive neurologic signs and symptoms present, where an epidural glucocorticosteroid injection is being considered	NASS	Recommended	Common	Should do
	VA/DoD	Recommended		
	TOP	Consider		

	NBHD	Only when recent lumbar nerve root lesions after careful consideration		
	EG	Recommend for evaluation of radicular symptoms		
	PRAM	Strongly endorsed		
	ACOEM	Moderately recommended		
	IHM	Recommended		
	ACP-APS	Only if patients are potential candidates for surgery or epidural steroid injection		
	UMHS	May be reasonable to perform		
	NBPN	Recommended, if severe persistent symptoms and referral to surgery is considered		
	ACR	Usually appropriate when underlying diseases present		
		May be appropriate when surgery or intervention candidate		
	ACP	suggested immediately when severe neurologic deficits		
Others				
EMG	EG	Not established	Inconsistent	None
	PRAM	Not recommend in the first four weeks of low back pain		
	ACOEM	Recommended where CT or MRI is equivocal and ongoing pain		
		Not recommended when no significant leg pain or numbness		
	IHM	Not recommended in first 4 weeks		
	UMHS	Should be performed If no improvement at ≥ 3 weeks and MRI not diagnostic		

Sensory nerve somatosensory evoked potentials (SEP)	NASS	Suggested as an adjunct to cross-sectional imaging	Inconsistent	None
	DLE-DWC	Not recommended to identify radiculopathy		
Discography	ACOEM	Moderately not recommended	Inconsistent	None
	DLE-DWC	May be indicated when unremitting pain > 3 months and non-invasive imaging not diagnostic		
	NBPN	Not recommended (do not contribute to diagnostic accuracy)		
Diagnostic medial branch block	APS	no recommendation	Inconsistent	None
	ACOEM	not recommended		

* Table 1 provides the extensive names of the included guidelines.

Journal of
Clinical Medicine

MDPI

Table S5. Therapeutic recommendations from guidelines for lumbar radicular pain.

Non-Invasive Treatment	Guide-line*	Specification	Consistency	Clinical Infer-ence
Bed rest	ACOEM	Not recommended	Inconsistent	None
	PARM	Not recommended (except for 2-4 days in severe cases)		
	IHM	Better not do, except 2-4 days in case of major sciatica		
	NHG	Can be considered for few days		
	NBPM	Not recommended, unless to relieve pain		
Physical activity	NBHD	Consider encouraging	Common	Should do
	PARM	Recommends remaining physically active		
	IHM	Recommend continuing usual everyday life activities		
	ACP-APS	Advice to remain active		
	NICE	Provide, promote and facilitate		
	NHG	Advice dosed physical activity		
	NBPN	Should be encouraged		
Educational care	NICE	Provide information on the nature sciatica	Consistent	Should do
	ICSI	Should receive		
	NHG	Discuss the condition		
	ACP-APS	Provide		
	NBPN	Provide information on the nature sciatica		
Multidisciplinary approach/re-habilitation program/ Psycho-logical therapy	NICE	Consider psychological therapy as part of a treatment package	Common	Could do
	KCE	Consider		
	UMHS	In patient at risk for chronic disability at 6 weeks and disabled		
	NBPN	Consider when risk of becoming chronic and psycho-social risk factors		
Alternative medicine				
Acupuncture	ICSI	Should be considered	Inconsistent	None
	PARM	Recommended in acute pain		
	ACOEM	Not recommended		
	IHM	Not effective		
	NICE	Do not offer		
	KIOM	Recommended		
	NBPN	Not recommended		
Manual therapies				

traction	NASS	Insufficient evidence to recommend	Inconsistent	None
	KCE	Do not offer		
	PRAM	Not recommended		
	ACOEM	Moderately not Recommended		
	DLE-DWC	Not recommended		
	NICE	Do not offer		
	NBPN	Cannot be recommended		
	APTA	Uncertain, a subgroup will benefit		
manipulation/mobilisation/soft-tissue techniques	NASS	Option for symptomatic relief.	Inconsistent	None
		No evidence for improve functional outcomes		
	KCE	Consider		
	ICSI	Should be considered		
	NBHD	Consider joint mobilization		
	PARM	Suggested in acute pain		
	ACOEM	Recommended when no neurological deficit		
	DLE-DWC	Generally accepted and well established intervention		
	IHM	Contraindicated		
	NICE	Consider only as part of a treatment package		
	NHG	Not recommended		
	CCGI	Conditionally recommended > 3 months pain		
	NBPN	Manipulation should not be used in the acute phase, could be considered as short-term pain relief when persistent symptoms		
	APTA	Should consider		
Message	PRAM	Not recommended	Inconsistent	None
	ACOEM	Could do		
	IHM	Do not do		
	NBPN	Not recommended		
Devices (e.g. belts, corset, foot orthotics etc.)	NICE	Do not offer belts, corsets, foot orthotics, rocker sole shoes	Consistent	Do not do
	KCE	Do not offer belts or corsets, foot orthics, rocker sole shoes		
	ACOEM	Shoe insoles and lifts are not recommended		
	ACOEM	Kinesiotaping and taping are not recommended		
	NBPN	Belts and corset are not recommended		
Exercise/physical therapies	KCE	Consider an exercise program	Consistent	Could do
	ACOEM	Moderately Recommended		

	NBHD	Weak/conditional recommendation		
	CCGI	Conditional recommendation for patients with chronic (> 3 months) leg pain		
	NBPN	Can be used as a tool to activate inactive patients		
	APTA	Can consider flexion exercises		
Electrotherapies *TENS/PENS/interferential therapy*	NICE	Do not offer TENS/PENS/interferential therapy	Consistent	Do not do
	KCE	Do not offer TENS/PENS/interferential therapy		
	PRAM	TENS not recommended		
	ACOEM	TENS/PENS generally not recommended, only recommended for select use		
	IHM	TENS not useful		
	NBPN	Not recommended		
Therapeutic ultrasound	KCE	Do not do	Inconsistent	None
	PARM	Recommended		
	NICE	Do not offer		
Heat/Cold/Infrared therapies	ICSI	Heat and Cold therapies may be used for pain relief	Inconsistent	None
	PRAM	Heat not recommended in acute pain		
	UMHS	Heat may be reasonable to perform in initial visit		
	ACOEM	No recommendation for infrared therapy		
**Pharmacological interventions**				
Paracetamol	KCE	Do not routinely offer	Inconsistent	None
	PARM	Recommended in acute pain		
	ACOEM	Recommended when NSAIDs are contraindicated		
	IHM	Could be useful to reduce pain		
	UMHS	Opinion of expert panel		
	NHG	Offer as first choice		
	NBPN	Start with		
Non-steroidal anti-inflammatory drugs (NSAID)	KCE	Consider	Inconsistent	None
	ICSI	May be used considering the side effect		
	PARM	suggested for acute pain		
	ACOEM	Strongly recommended		
	IHM	Could be useful to reduce pain		
	NHG	Give as step 2 only if Paracetamol is effective		
	NBPN	Do not use (evidence of no effect and risk of side-effects)		
Opioids	NICE	Consider Tramadol only for acute rescue therapy<br>Do not start Tramadol for long term use in non-specialist setting,<br>Do not start Morphine in non-specialist setting	Inconsistent	None
	KCE	Do not routinely offer for managing chronic pain		

	PARM	Light opioid recommended in acute pain in combination with paracetamol as alternative when NSAIDs or paracetamol alone do not control pain		
	IHM	Strong recommendation. Could be useful to reduce pain		
	NHG	Consider strong opioids like Morphine/ Fentanyl in chronic pain		
	NBPN	Consider Tramadol for acute strong pain, but only for short periods		
Paracetamol + opioids	IHM	Can be an effective alternative when NSAIDs or PCM alone do not control pain	Inconsistent	None
	NHG	Not recommended		
	NBPN	Start if no satisfactory effect with paracetamol alone		
Anti-epilepticum	KCE	Do not offer	Inconsistent	None
	VA/Dod	No recommendation for or against		
	PARM	Recommended in acute pain		
	ACOEM	Not recommended for chronic radicular pain syndromes		
	NASS	insufficient evidence to make a recommendation for or against Gabapentin		
	NICE	Offer gabapentin or Pregabalin<br>Do not start in non-specialist setting: levetiracetam, lamotrigine,<br>topiramate, oxcarbazepine, lacosamide		
	NHG	Consider Gabapentine besides low dose of TCA, if TCA is not effective, or has side effect of is contra indicated		
Muscle relaxants	KCE	Do not offer	Inconsistent	None
	ICSI	May be used as a short-term option		
	PARM	Recommended in acute pain		
	ACOEM	Recommended for acute radicular pain		
	IHM	Could be useful to reduce pain		
	NHG	Benzodiazepines are not recommended		
	NBPN	Evaluate but only for a short period		
Antidepressants	NASS	Insufficient evidence for Amitriptyline	Inconsistent	None
	NICE	Offer amitriptyline or duloxetine<br>Do not start venlafaxine in non-specialist setting		
	KCE	Do not offer SSRI, TCA or SNRI		
	ACOEM	SSRI is not recommended.<br>No recommendation for TCA/SNRI		
	NHG	TCA/SNRI could be considered		
Corticosteroids	VA/Dod	Recommended against offering	Inconsistent	None

	PARM	Recommended short-term for patients with acute pain		
	ACOEM	Recommended for treatment of acute and subacute radicular pain		
		Not recommended for treatment of chronic radicular pain		
	DLE-DWC	Not generally recommended		
	NHG	Not recommended		
	ACP-APS	Not recommended		
Antibiotics	KCE	Do not offer	Inconsistent	None
	ACOEM	No recommendation		
Cannabis	NICE	Do not start in non-specialist settings	Consistent	Do not do
	NHG	Not recommended in primary care		
Invasive Treatments				
Surgery	NASS	Percutaneous/Automated discectomy may be suggested	Common	Could do
	APS	Consider offering discectomy		
	NHG	Consider after 12 weeks of conservative therapy		
	DLE-DWC	Discectomy/Laminectomy/Foramenotomy/Facetectomy/Chemonucleolysis are indicated		
	IHM	Discectomy effective when no improvement with conservative therapy		
		laser/Percutaneous discectomy must be considered experimental		
	ACOEM	Lumbar discectomy is moderately recommended after 4 to 6 weeks to speed recovery		
		Automated percutaneous discectomy and Laser discectomy not recommended		
	NBPN	Acute only for cauda equina syndrome		
		Discectomy can be considered after 12 weeks (preferably before 8 months) when persistent severe symptoms		
Injection therapies *Epidural injections*	NICE	Consider acute and severe sciatica	Inconsistent	None
	NASS	Transforaminal epidural steroid injection are recommended		
		Interlaminar epidural steroid injections may be considered		
	KCE	Consider in (sub)acute (at least 2-3 weeks) and severe pain		
	VA/Dod	Suggested for the very short-term effect		

		Recommended against offering for the long-term reduction of pain		
	ICSI	May be used as an adjunct treatment to assist with short-term pain relief		
	NBHD	Weak/conditional recommendation against extra-foraminal glucocorticoid injection		
	PRAM	Recommended		
	ACOEM	Recommended as an option		
	ACP	Weak recommendation		
	NHG	Not recommended in first line		
	NBPN	Can be tried in awaiting surgery		
	APS	Consider offering when prolapsed lumbar disc		
Referral			Consistent	should do
	PRAM	Immediate referral for surgical evaluation when steppage		
	IHM	To neurophysiological when etiological/ level diagnosis are uncertain,<br>Prognostication information required or monitor objectively<br>Surgical evaluation < 1 month when neurological symptoms worsening<br>Or > 1 month when sciatica is important and disabling		
	NHG	Neurologist evaluation when 1-3 months sciatica<br>Neurologist evaluation when 1-3 months sciatica<br>1-2 day if sever pain despite maximal medication		

* Table 1 provides the extensive names of the included guidelines.
